# Molecular cytogenetic analyses reveal extensive chromosomal rearrangements and novel B chromosomes in *Moenkhausia* (Teleostei, Characidae)

**DOI:** 10.1590/1678-4685-GMB-2020-0027

**Published:** 2020-11-06

**Authors:** Cristiano Neves do Nascimento, Waldo Pinheiro Troy, José Carlos Pansonato Alves, Margarida Lima Carvalho, Claudio Oliveira, Fausto Foresti

**Affiliations:** ^1^ Universidade Estadual Paulista - UNESP, Instituto de Biociências, Departamento de Biologia Estrutural e Funcional, Botucatu, SP, Brazil.; ^2^ Universidade do Estado de Mato Grosso - UNEMAT, Departamento de Ciências Biológicas, Tangará da Serra, MT, Brazil.; ^3^ Centro Universitário Toledo - UNITOLEDO, Araçatuba, SP, Brazil.; ^4^ Universidade Federal do Acre - UFAC, Centro de Ciências Biológicas e Naturais, Rio Branco, AC, Brazil.

**Keywords:** Chromosomal mapping, repetitive DNA, multigenic families, fish karyotypes, supernumerary chromosomes

## Abstract

The cytogenetic characteristics of five fish species of the *Moenkhausia* are described*,* based on the analysis of specimens collected in different headwater. All the species analyzed presented 2n=50 chromosomes. The C-banding revealed a similar distribution pattern of heterochromatic blocks in all the species, except *Moenkhausia nigromarginata*. The 5S rDNA sites were distributed on multiple chromosome pairs in all five species. Single and multiple histone H1 sites were observed in all the species, and histone H1 was shown to be co-located with the 18S rRNA gene in a single chromosome pair. The U2 snDNA gene was distributed at multiple sites in all the *Moenkhausia* species. The presence of B microchromosomes was confirmed in *Moenkhausia forestii*, while individuals of the three study populations of *Moenkhausia oligolepis* presented three morphologically distinct types of B chromosome. The chromosomal mapping of the 18S rDNA sites using the FISH technique revealed signals in the B chromosomes of *M*. *forestii*, while clusters of the H1 histone and U2 snDNA genes were found in the B chromosomes of *M. forestii* and *M. oligolepis*. The classical and molecular cytogenetic markers used in this study revealed ample variation in the *Moenkhausia* karyotypes, reflecting the dynamic nature of the chromosomal evolution.

## Introduction

*Moenkhausia* Eigenmann, 1903 is one of the most speciose fish genera of the family Characidae, with more than 100 valid species distributed amply in the rivers and streams of the Neotropical region (Fricke *et al.*, 2019). The greatest diversity of this group can be found in the bodies of water of the Amazon and Guiana basins (Lima *et al.*, 2003). Given their ample geographical distribution and morphological diversity, the *Moenkhausia* species represent an interesting group for evolutionary (Benine *et al.*, 2009; Mirande 2010; Oliveira *et al.*, 2011; Mariguela *et al.*, 2013), taxonomic (Benine *et al.*, 2007; Marinho and Langeani 2010; Pastana and Dagosta 2014; Reia *et al.*, 2019), ontogenetic (Walter 2012), and cytogenetic studies (Portela *et al.*, 1988; Foresti *et al.*, 1989; Portela-Castro *et al.*, 2001; Dantas *et al.*, 2007; Hashimoto *et al.*, 2012b; Scudeler *et al.*, 2015; Utsunomia *et al.*, 2016).

Cytogenetic studies of *Moenkhausia* have shown that its species have relatively well-conserved diploid numbers, with either 2n=48 or 2n=50 chromosomes, and a predominance of metacentric and submetacentric chromosomes (Portela-Castro and Júlio–Júnior 2002; Dantas *et al.*, 2007). Despite the apparent conservation of the karyotype, there is considerable variation in the distribution of the Nucleolus Organizing Regions (NORs) and heterochromatic blocks, which can be observed not only among, but also within populations, while two species, *Moenkhausia intermedia* and *Moenkhausia sanctaefilomenae* also have B chromosomes in their complements (Portela *et al.*, 1988; Foresti *et al.*, 1989; Portela-Castro *et al.*, 2001; Dantas *et al.*, 2007). Remarkably, these supernumerary elements vary considerably in their morphology and distribution, and are restricted to only males in a population of *M*. *sanctaefilomenae* (Portela *et al.*, 1988; Foresti *et al.*, 1989; Portela-Castro and Júlio–Júnior 2002; Dantas *et al.*, 2007).

Although the karyotypic characteristics of the *Moenkhausia* species are relatively well-known, it is important to note that the majority of the data compiled up to now have been obtained from only a few species. This highlights the need to analyze other species of this genus, in order to better understand the mechanisms involved in the speciation process. To amplify the *Moenkhausia* database and the understanding of these mechanisms, the present study applied both classical and molecular cytogenetic approaches to the analysis of the karyotypes of five species, *Moenkhausia cosmops*, *M*. *forestii*, *M*. *nigromarginata*, *M*. *oligolepis*, and *Moenkhausia* sp. n.

## Material and Methods

### Sampling localities and cytogenetic analyses

**Figure 1:**
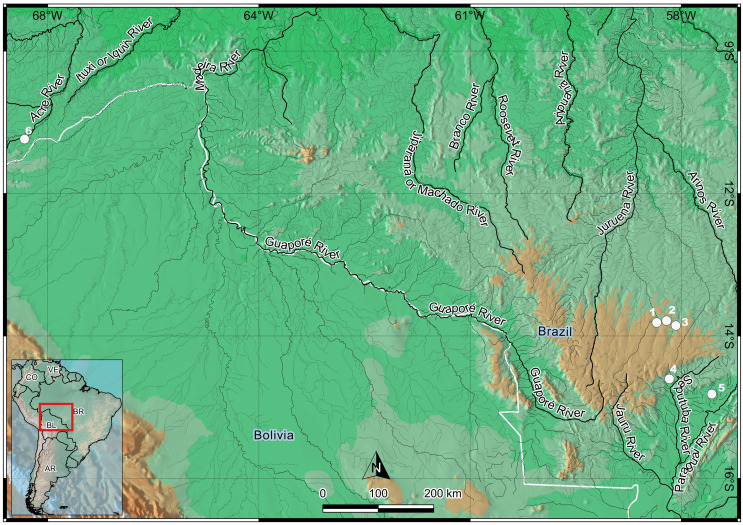
Location of the collecting localities of the *Moenkhausia* specimens analyzed in the present study: **(1)** Verde River (*M*. *cosmops* and *M*. *nigromarginata*); **(2)** Membeca River (*Moenkhausia* sp. n. and *M*. *nigromarginata*); **(3)** Sangue River (*M*. *oligolepis*); **(4)** Sapo Stream (*M*. *forestii*); **(5)** Corredeira Stream (*M*. *oligolepis*); and **(6)** Xapuri River (*M*. *oligolepis*).

For the analyses presented here, representatives of five species of the genus *Moenkhausia* were collected from rivers and headwater streams of the basins of the Amazon River and the upper Paraguay River (Figure 1), in the Brazilian states of Acre and Mato Grosso, respectively. The samples (Table 1) were collected in accordance with the procedures mandated by Brazilian environmental legislation (authorization for specimen collection: MMA/IBAMA/SISBIO – number 3245). The collection, storage, and analysis of the samples all followed international protocols on animal experiments, as authorized by the São Paulo State University (CEUA Protocol/IBB/UNESP – number 504). The specimens were identified and deposited in the collection of the UNESP Laboratory of Fish Biology and Genetics in Botucatu, São Paulo, Brazil (Table 1).

**Table 1:** – Specimens of *Moenkhausia* collected. LBP: Coleção de Peixes Laboratório de Biologia e Genética de Peixes, Instituto de Biociências, UNESP; F: females; M: male.

Species	Coordinates	Map points	Locality/City	LBP	Sample	
					M	F
*Moenkhausia cosmops*	13°38’34.77“S, 58°01’03.02”W	1	Verde River – Campo Novo do Parecis/MT	8164	2	2
*M. forestii*	14°33’24.43“S, 57°48’45.53”W	4	Ribeirão do Sapo – Tangará da Serra/MT	19532	4	8
*M. oligolepis*	14°48’08.33“S, 57°07’25.18”W	5	Corredeira Stream – Denise/MT	19530	4	4
	13°41’30.56“S, 57°42’23.28”W	3	Sangue River – Campo Novo do Parecis/MT	8527	3	4
	10°40’03.63“S, 68°15’43.61”W	6	Nameless Stream – Xapuri/AC	18576	3	2
*M. nigromarginata*	13°38’34.77“S, 58°01’03.02”W	1	Verde River – Campo Novo do Parecis/MT	19533	1	2
	13°36’43.82“S, 57°51’28.29”W	2	Membeca River – Campo Novo do Parecis/MT	8525	1	0
*Moenkhausia* sp. n.	13°36’43.82“S, 57°51’28.29”W			19531	4	1

Mitotic chromosome preparations were obtained from renal tissue and gills using the protocol proposed by Foresti *et al.* (1981) The metaphase chromosomes were examined under an optical photomicroscope (Olympus BX61) and the images were captured using an Olympus DP70 digital camera. The chromosome morphology was determined according to the ratio of the arms, as established by Levan *et al.* (1964), and the chromosomes were classified as metacentric (m), submetacentric (sm), subtelocentric (st) or acrocentric (a), and arranged in descending order of size in the assembly of the karyotypes. The NORs were stained with Silver nitrate following the technique proposed by Howell and Black (1980), and the C-banding was based on the protocol described by Sumner (1972).

### Amplification of repetitive DNAs and Fluirescence *in situ* Hybridization (FISH)

The telomeric sequences and the 5S and 18S rDNA, histone H1, and U2 snDNA genes were amplified by PCR (Polymerase Chain Reaction) from the total DNA of the *M*. *forestii* and *Moenkhausia* sp. n. specimens using the primers shown in Table S1. The probes were labeled with digoxigenin-11-dUTP or biotin-16-dUTP (Roche) in the secondary PCR reactions.

The repetitive sequences were mapped physically by the FISH technique, following Pinkel *et al.* (1986). The fluorescent signals were detected using anti-digoxigenina-rhodamine (Roche) for the probes marked with digoxigenin-11-dUTP, and FITC-avidin amplified with biotinylated anti-avidin (Sigma) for the probes labeled with biotin-16-dUTP. Following the fluorescent preparations, the chromosomes were counterstained with DAPI, and the metaphases were photographed under an epifluorescence photomicroscope (Olympus BX61), with the images being captured using an Olympus DP70 digital camera.

## Results

### Standard chromosome complements and repetitive DNA sequences

**Figure 2:**
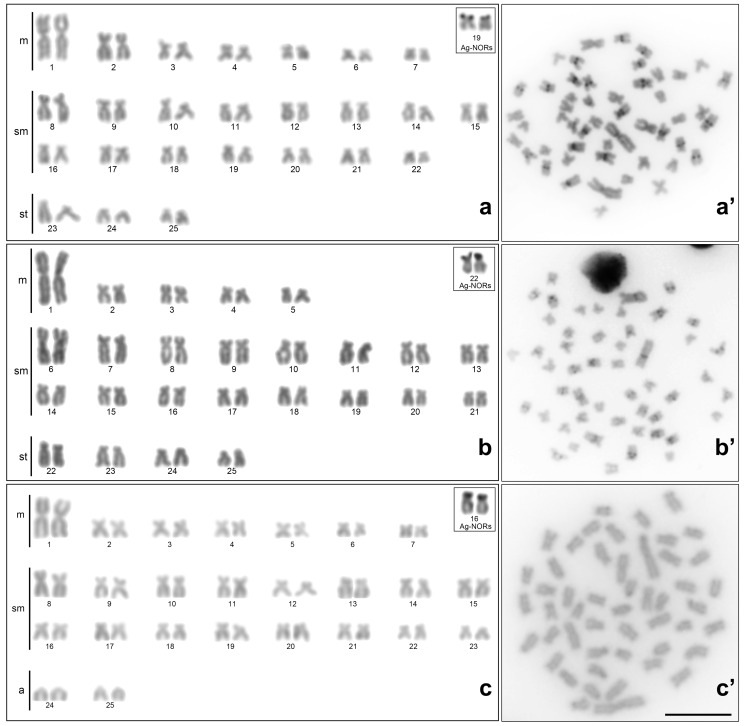
Karyotypes stained by 5% Giemsa (**a**, **b**, and **c**) and metaphases after C-banding (**a’**, **b’**, and **c’**) of **(a)** *M*. *cosmops*, **(b)** *Moenkhausia* sp. n., and **(c)** *M*. *nigromarginata*. The Ag-NORs are shown in the box. Scale bar = 10*μ*m.

**Figure 3:**
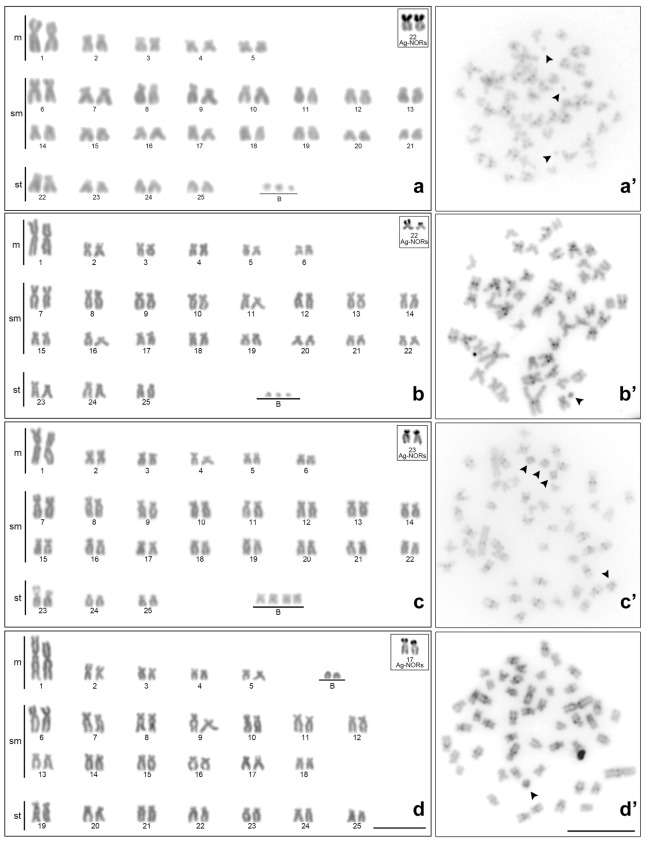
Karyotypes stained by 5% Giemsa (**a**, **b**, and **c**) and the metaphases after C-banding (**a’**, **b’**, and **c’**) of **(a)** *M*. *forestii*; **(b)** *M*. *oligolepis* (Sangue River); **(c)** *M*. *oligolepis* (Corredeira Stream), and **(d)** *M*. *oligolepis* (Xapuri River). The B chromosomes (arrowheads) are shown in the insert. The Ag-NORs are represented in the box. Scale bar = 10 *μ*m.

The karyotypic analyses of the specimens of *Moenkhausia cosmops*, *M*. *forestii*, *M*. *nigromarginata*, *M*. *oligolepis,* and *Moenkhausia* sp. n. revealed a diploid chromosome number of 2n=50 in all the species (Figures 2 and 3), albeit with some variation in the karyotype formula (Table 2). No chromosomal polymorphisms related to sex were detected in any of the species.

**Table 2:** Cytogenetic data found in the species or populations of *Moenkhausia* analyzed in this study.

Species	(Map point) Locality	Diploid Number	Karyotypic Formula	FN	B-Chromosomes
*Moenkhausia cosmops*	**(1)** Verde River	50	14m+30sm+6st	100	-
*M. forestii*	**(4)** Ribeirão do Sapo	50	10m+32sm+8st	100	0-3
*M. oligolepis*	**(5)** Corredeira Stream	50	12m+32sm+6st	100	0-4
	**(3)** Sangue River				0-3
	**(6)** Xapuri River	50	10m+26sm+14st		0-2
*M. nigromarginata*	**(2)** Membeca River	50	14m+32sm+4a	96	-
	**(1)** Verde River				-
*Moenkhausia* sp. n.	**(2)** Membeca River	50	10m+32sm+8st	100	-

The heterochromatin was distributed in a similar pattern in the chromosomes of *M. cosmops*, *M*. *forestii*, *M*. *oligolepis,* and *Moenkhausia* sp. n., with heterochromatic blocks being distributed in the centromeric or pericentromeric regions of the chromosomes (Figures 2 and 3). A different pattern was observed in *M*. *nigromarginata*, however, with the heterochromatin being distributed in small centromeric blocks in the acrocentric chromosomes and in the terminal regions of some chromosomes (Figure 3c’). In addition, Ag-stained NORs were detected in the terminal position of the short arms of submetacentric or subtelocentric chromosomes in all the species analyzed (Figure 2 and 3).

**Figure 4:**
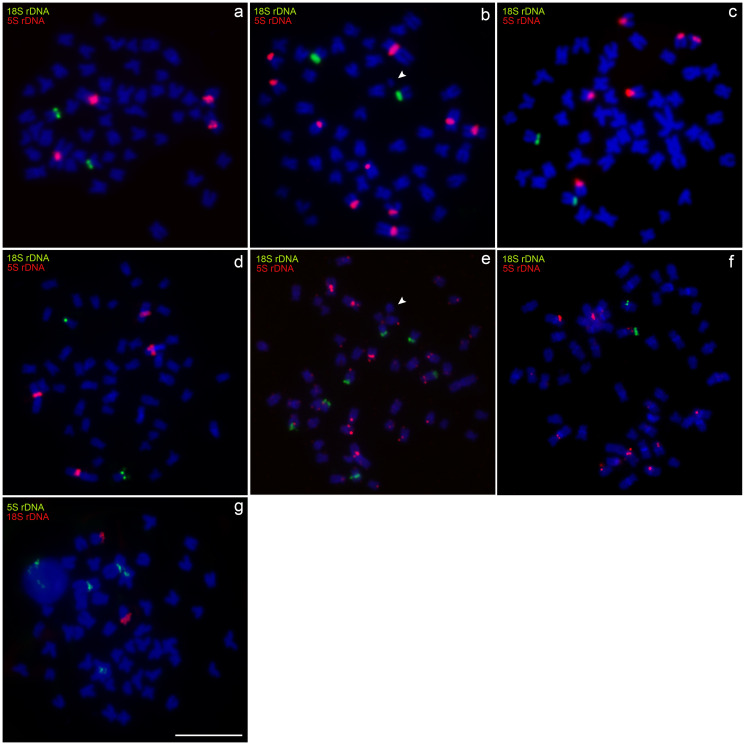
Metaphases mapped by double-FISH with 18S and 5S rDNA probes in: **(a)** *M*. *cosmops*; **(b)** *M*. *forestii*; **(c)** *M*. *nigromarginata*; **(d)** *Moenkhausia* sp. n*.*; **(e)** *M*. *oligolepis* (Xapuri River); **(f)** *M*. *oligolepis* (Corredeira Stream), and **(g)** *M*. *oligolepis* (Sangue River). The B chromosomes are indicated by arrowheads. Scale bar = 10 *μ*m.

Each species and population presented a unique set of characteristics in relation to the location and distribution of the 5S rDNA sites (Figure 4). In *M*. *cosmops*, the 5S rDNA gene was mapped in the pericentromeric region of chromosome pairs 1 and 2 (Figure 4a), while in *M*. *forestii*, clusters of 5S rDNA were identified in centromeric positions in pairs 1, 2, 6, 8, and 10 (Figure 4b). In the *M*. *nigromarginata* specimens from the Membeca River, the 5S rDNA clusters were observed in a centromeric position in the acrocentric pairs 24 and 25, while the specimens from the Verde River had an additional 5S rDNA cluster in the short arms of pair 19 (Figure 4c). *Moenkhausia* sp. n. had 5S rDNA clusters in the pericentromeric region of chromosome pairs 1 and 6 (Figure 4d).

In *M*. *oligolepis*, furthermore, the position and distribution of the 5S rDNA sequences varied among the three populations analyzed. In the specimens from the Xapuri River, the sequences were dispersed in the pericentromeric or centromeric portions of 21 chromosomes (Figure 4e). In the population from the Corredeira Stream, the specimens had 5S rDNA sites scattered in the centromeric or pericentromeric regions of up to 17 chromosomes, including pairs 1 and 7, as well as centromeric signals in the NOR-bearing chromosomes (Figure 4f). In the specimens from the Sangue River, by contrast, only four signals were found, in a centromeric position in chromosome pairs 1 and 7 (Figure 4g). Despite all this variation in the distribution of the 5S rDNA sequences, fluorescent signals were observed in the chromosomes of pairs 1 and 7 in specimens from all three populations. The 5S rDNA clusters in the pericentromeric region of this species coincided with the distribution of the blocks of constitutive heterochromatin.

**Figure 5:**
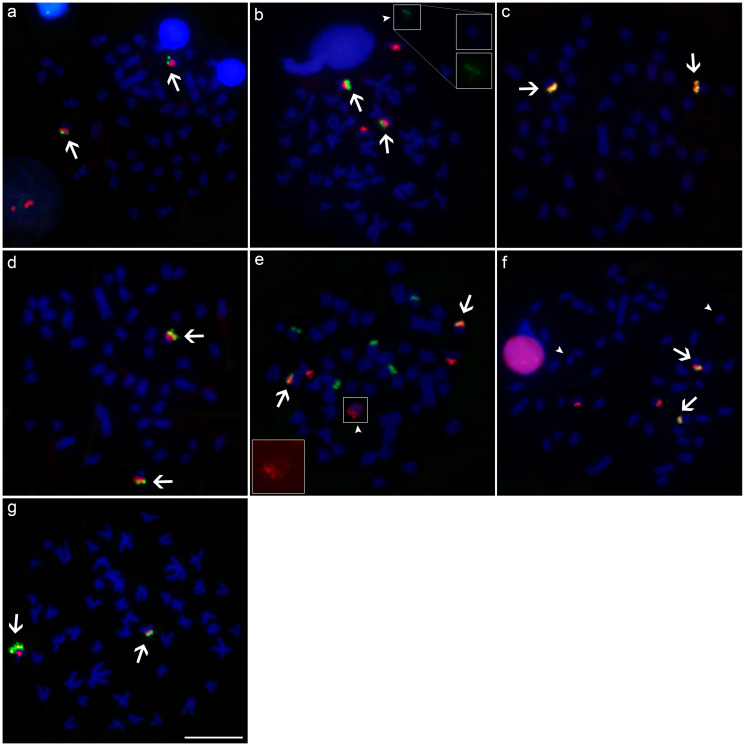
Metaphases mapped by double-FISH with H1 histone (red) and 18S rDNA (green) probes in: **(a)** *M*. *cosmops*; **(b)** *M*. *forestii*; **(c)** *M*. *nigromarginata*; **(d)** *Moenkhausia* sp. n.; **(e)** *M*. *oligolepis* (Xapuri River); **(f)** *M*. *oligolepis* (Corredeira Stream), and **(g)** *M*. *oligolepis* (Sangue River). Chromosome synteny between markers is indicated by the arrows, while the B chromosomes are shown by the arrowheads. Scale bar = 10 *μ*m.

The results of the double FISH with H1 and 18S rDNA probes indicated the co-location of sites in the terminal region of the short arm of a single chromosome pair in almost all the species or populations examined (Figure 5). The exceptions were *M*. *forestii* and two *M*. *oligolepis* populations (Figure 5b, e, f). In *M*. *forestii* and the Corredeira population of *M*. *oligolepis*, the H1 sites were co-located with 18S rDNA in only a single chromosome pair. In the Xapuri *M*. *oligolepis* population, by contrast, 18S rDNA sites were observed in seven chromosomes (Figure 5e).

**Figure 6:**
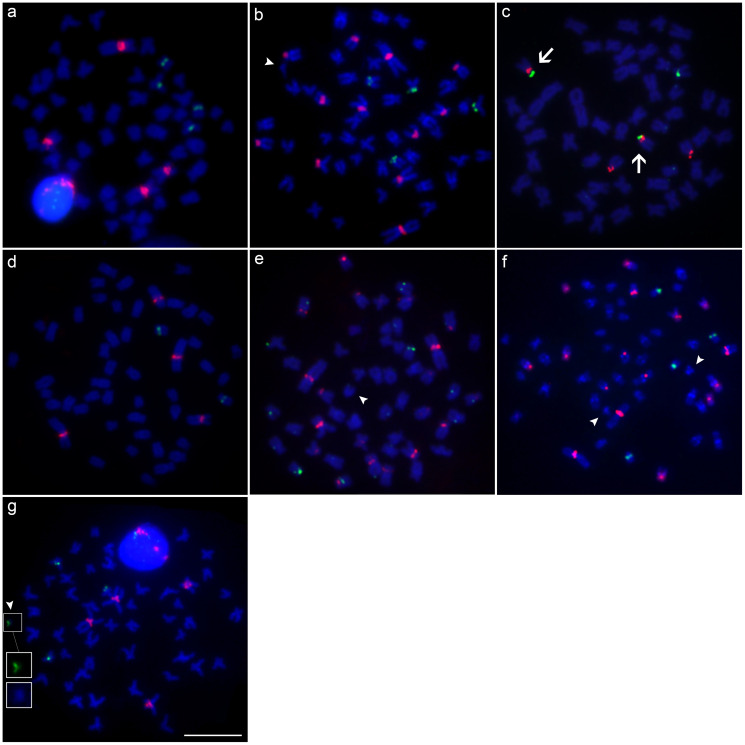
Metaphases mapped by double-FISH with 5S rDNA (red) and U2 snDNA (green) probes in: **(a)** *M*. *cosmops*; **(b)** *M*. *forestii*; **(c)** *M*. *nigromarginata*; **(d)** *Moenkhausia* sp. n.; **(e)** *M*. *oligolepis* (Xapuri River); **(f)** *M*. *oligolepis* (Corredeira Stream), and **(g)** *M*. *oligolepis* (Sangue River). Chromosome synteny between markers is indicated by the arrows, while the B chromosomes are shown by the arrowheads. Scale bar = 10 *μ*m.

Physical mapping showed that the U2 snDNA gene occupies multiple sites in all the *Moenkhausia* species examined. In *Moenkhausia* sp. n., the sites are located in chromosome pairs 4 and 19 (Figure 6d), while in *M*. *nigromarginata*, they were observed in pairs 6, 22, and 24, being syntenic with 5S rDNA in pair 24 (Figure 6c). In *M*. *cosmops*, the U2 snDNA gene was observed in multiple chromosome pairs (Figure 6a), while in *M*. *forestii*, U2 sites were identified in two submetacentric pairs (Figure 6b). In *M. oligolepis*, the mapping of U2 revealed distinct patterns in all three study populations, with three chromosome pairs being tagged in the Sangue population and four in the Xapuri and Corredeira populations (Figure 6g).

**Figure 7:**
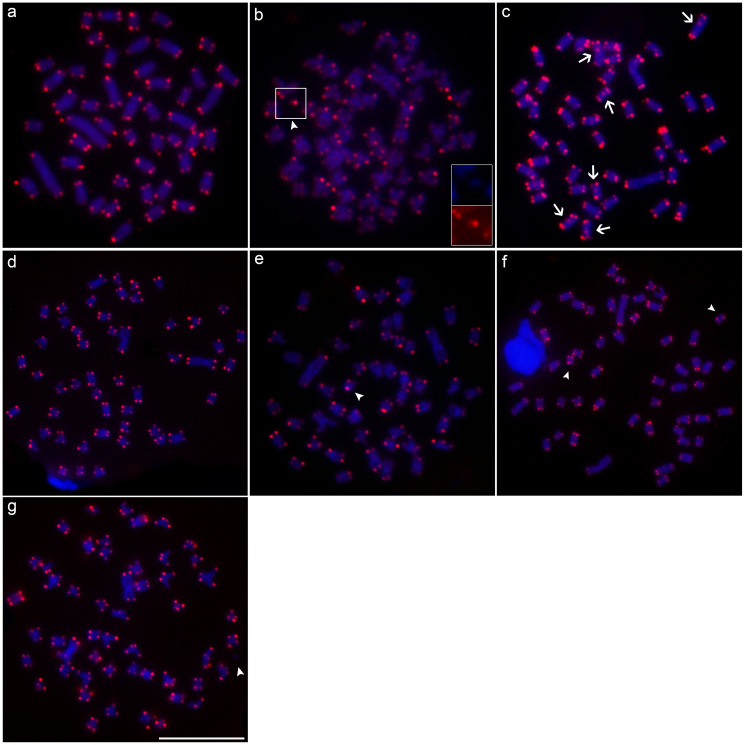
Metaphases mapped by FISH with the telomeric probe (TTAGGG)_n_ in: **(a)** *M*. *cosmops*; **(b)** *M*. *forestii*; **(c)** *M*. *nigromarginata*; **(d)** *Moenkhausia* sp. n.; **(e)** *M*. *oligolepis* (Xapuri River); **(f)** *M*. *oligolepis* (Corredeira Stream), and **(g)** *M*. *oligolepis* (Sangue River). Interstitial signals are indicated by the arrows, while the B chromosomes are shown by the arrowheads. Scale bar = 10 *μ*m.

The hybridization with telomeric probes demonstrated the typical pattern of telomeric signals in the terminal position of all the chromosomes of all five species analyzed. Interstitial Telomeric Sequences (ITSs) were observed only in three chromosome pairs of *M*. *nigromarginata* (Figure 7c).

### B chromosomes

In addition to the standard complement of chromosomes, that is, the A chromosomes, B chromosomes were observed in *M*. *forestii* and *M*. *oligolepis* (Figure 3). One to three B microchromosomes were identified in six of the 11 specimens of *M*. *forestii* analyzed (Figure 3a). In *M*. *oligolepis*, the B chromosomes varied considerably in number and morphology among the three populations sampled (Figures 3b, c, d). The *M*. *oligolepis* specimens from the Sangue River had 0–3 B microchromosomes, or B_*micro*_ (Figure 3b), while those of the Corredeira population had 0–4 metacentric B chromosomes (B_*m*_) of small size, which were similar to the smallest metacentric pair of the standard complement (Figure 3c). In the population from the Xapuri River, individuals with 0–2 acrocentric B chromosomes (B_*ac*_) were observed (Figure 3d). These chromosomes varied in frequency at both intra- and inter-individual levels (Table S2). The supernumerary elements in *M*. *forestii* and *M*. *oligolepis* also presented distinct heterochromatic patterns, with euchromatic and fully or partially heterochromatic chromosomes. The individuals from the Xapuri River that had B_*ac*_ chromosomes presented only partially heterochromatic chromosomes (Figure 3d’).

**Figure 8:**
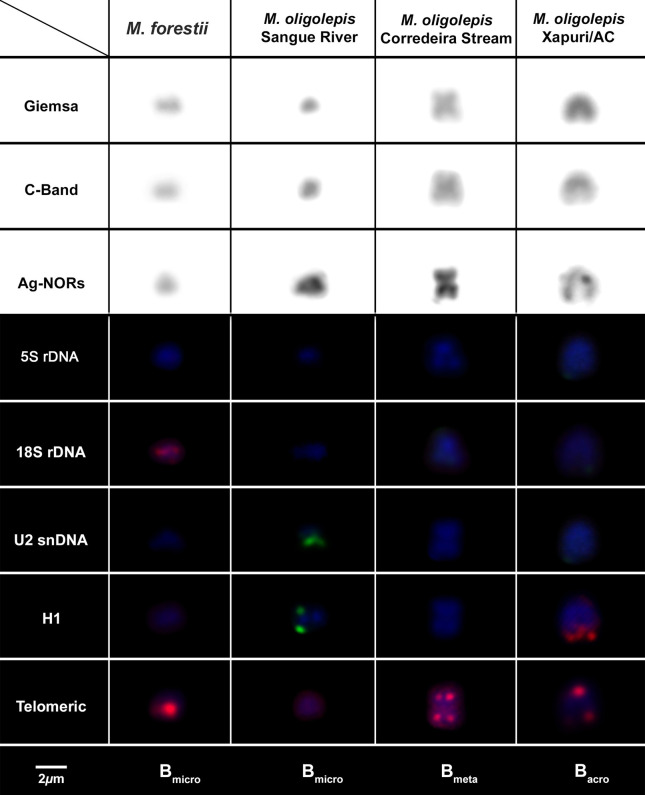
The B chromosomes of the *Moenkhausia* species analyzed in the present study after the application of different cytogenetic techniques.

The chromosomal mapping of the 18S rDNA, histone H1, and U2 snDNA sites using the FISH technique revealed fluorescent signals in the B_*micro*_ of *M*. *forestii* (Figures 5b and 8). Clusters of the H1 histone gene were found in the terminal portion of the long arm in the B_*ac*_ chromosomes of *M*. *oligolepis* in individuals from the Xapuri River and in B_*micro*_ chromosomes of the Sangue River population (Figure 8). Fluorescent signals of the U2 snDNA gene were also observed in the B_*micro*_ chromosomes of the Sangue River population (Figures 6g and 8). However, no cytogenetic markers were identified in the B_m_ chromosomes in individuals of the Corredeira population. The telomeric probe used in the present study, (TTAGGG)_n_, did not indicate interstitial signals in any of the B chromosomes of *M*. *forestii* or *M*. *oligolepis* (Figure 7 and 8).

## Discussion

The diploid number (2n=50) of the *Moenkhausia* taxa analyzed in the present study was consistent with the numbers recorded in other *Moenkhausia* species, indicating a conserved karyotype, with a predominance of bi-armed chromosomes (Portela *et al.*, 1988; Foresti *et al.*, 1989; Portela-Castro *et al.*, 2001; Dantas *et al.*, 2007). Despite the constant diploid number and the minimal variation in the number of chromosome arms (96–100), differences were observed in the karyotype formula among the *Moenkhausia* species and populations studied here. Variations in the formula with a constant diploid number may be related primarily to non-Robertsonian structural rearrangements, such as inversions or translocations (Schubert 2007). It seems reasonable to conclude that these types of rearrangement, which are common in many different fish orders and families (Galetti *et al.*, 2000; Sato *et al.*, 2004; Silva *et al.*, 2013; Takagui *et al.*, 2014), have played a prominent role in the karyotype differentiation of *Moenkhausia*.

The observation of Interstitial Telomeric Sites (ITSs) in *M*. *nigromarginata*, despite the conservation of the typical *Moenkhausia* diploid number, indicates that the sequences have moved through non-Robertsonian structural rearrangements. In turn, the notable absence of ITSs from the chromosomes of *M*. *cosmops*, *M*. *forestii*, *M*. *oligolepis*, and *Moenkhausia* sp. n. indicates that any such rearrangement either did not involve the movement of large sequences of telomeres or only involved pericentromeric inversions.

Considerable variation was found in the distribution of the 5S rDNA sites in the *Moenkhausia* species, ranging from only four chromosomes in some populations to up to 21 chromosomes in others, reflecting the intense evolutionary dynamics of these sites. However, the interstitial location of these sites is a conserved pattern. Some authors have suggested that the interstitial position occupied by the 5S rDNA sites on the chromosome guarantees greater stability in comparison with the terminal region, and would thus help to avoid major genomic changes that would result in the dispersal of the sequences (Mantovani *et al.*, 2005; Nakajima *et al.*, 2012). In this context, the considerable diversification observed in the present study may be related to the association of these sites with transposable elements, which is also assumed to occur in other organisms (Nakajima *et al.*, 2012; Silva *et al.*, 2013; Silva *et al.*, 2014).

On the other hand, the chromosomal mapping of the U2 snDNA gene revealed a highly conserved distribution pattern among the different species, which is consistent with the general pattern of this cistron in closely related species (Cabral-de-Mello *et al.*, 2012; Utsunomia *et al.*, 2014). Interestingly, the 5S rDNA and U2 snDNA sites were located in synteny in some *Moenkhausia* species, implying a co-location pattern in these repetitive DNA sequences. Hashimoto *et al.* (2011; 2012a) suggested that the co-location of the histone and ribosomal cistrons in other fish genera may confer a selective advantage and would likely be related to the general clustering tendency of housekeeping genes, i.e., cistrons with high expression rates that are required for basic cellular functions. The results of the present study indicate a conserved association of the 18S rRNA and histone genes in the study species. This appears to be an ancestral feature of the genus *Moenkhausia*, given that it has remained unaltered throughout the evolutionary history of this group, further supporting the hypothesis of the clustering of housekeeping genes.

In addition to these broad similarities among the karyotypes of the five *Moenkhausia* species analyzed here, two species presented B chromosomes in a considerable variety of morphological configurations, representing the first description of these elements in *Moenkhausia forestii* and *M*. *oligolepis*. Supernumerary B chromosomes have been described in two *Moenkhausia* species, being described as small B chromosomes in *M. intermedia* and microchromosomes in *M*. *sanctaefilomenae* (Portela *et al.*, 1988; Foresti *et al.*, 1989; Portela-Castro *et al.*, 2001; Portela-Castro and Júlio–Júnior 2002; Dantas *et al.*, 2007; Hashimoto *et al.*, 2012b). The remarkable diversity of the morphology of these elements was further confirmed in the present study, with B_*micro*_, B_*m*_ and B_ac_ morphotypes being identified in different populations of *M*. *oligolepis*. Morphologically distinct B chromosomes have been described in a range of Neotropical fishes, in which microchromosomes are the most frequent type (Carvalho *et al.*, 2008; Oliveira *et al.*, 2009). This morphological polymorphism may be the result of chromosomal rearrangements, the accumulation of heterochromatin or the dynamics of the processing of repetitive DNA sequences (Camacho 2005). In fact, the heterochromatin plays a significant role in the diversification of the B chromosome in species of the family Characidae (Foresti *et al.*, 1989; Salvador and Moreira-Filho 1992; Poletto *et al.*, 2010; Voltolin *et al.*, 2010; Hashimoto *et al.*, 2012b).

In this specific case, the heterochromatic patterns observed in the B chromosomes of *M*. *forestii* and *M*. *oligolepis* may provide a clue to the considerable amount of repetitive DNA found in these two species. In a meticulous study, Utsunomia *et al.* (2016) observed two distinct C-banding patterns in the B chromosomes of *M*. *sanctaefilomenae*. In this same species, but in different other local population, Scudeler *et al.* (2015) observed an apparent similarity between the heterochromatin present in the B chromosome and that found in the standard complement (A chromosomes), and suggested a possible “silencing” effect of this heterochromatin.

Repetitive DNA sequences, such as rDNA, satellites, and histone genes have been found in the B chromosomes of a range of different fish species, including *Astyanax scabripinnis* and *Astyanax paranae* (Mestriner *et al.*, 2000; Silva *et al.*, 2014), *Prochilodus lineatus* (Artoni *et al.*, 2006), and *Astatotilapia latifasciata* (Poletto *et al.*, 2010; Fantinatti *et al.*, 2011). Nucleolar activity, identified by the Ag-NOR technique, has also been observed in the B chromosomes of *Moenkhausia sanctaefilomenae* (Hashimoto *et al.*, 2012b; Utsunomia *et al.*, 2016). It is interesting to note that, in these studies, the presence of these sequences in the B chromosomes was used as evidence of the identity of the probable ancestral chromosome that gave rise to this supernumerary element in the karyotype of the carrier species. The present study provides the first record of the occurrence of snDNA U2 genes in a fish microchromosome, a phenomenon reported previously in the grasshopper *Abracris flavolineata* (Bueno *et al.*, 2013). In addition, H1 histone clusters were observed in the B_micro_ and B_ac_ chromosomes of *M*. *oligolepis*, which indicates the presence of homologies between these B chromosomes and the possibility of a joint location of the histone H1 and 18S rDNA sites, reflecting the origin of these B chromosomes from ancestral A chromosomes. Even so, it is not entirely unlikely that the presence of these sequences in the B chromosomes is related to transposition events that are not directly linked to any homology. Given this, a more detailed investigation of the B chromosomes identified in the present study, based on more specific approaches, such as microdissection and chromosome painting, as well as massive sequencing, should provide more conclusive evidence on the origin, composition, and evolution of these supernumerary elements in the genus *Moenkhausia*.

## Supplementary material

The following online material is available for this article:

Table S1 – List of primers used in the PCR amplifications.

Table S2 – Observed variation in the number of B chromosomes in *Moenkhausia*.
