# Supplementary material for: Molecular cytogenetic analyses reveal extensive chromosomal rearrangements and novel B chromosomes in Moenkhausia (Teleostei, Characidae)
Source: Genet Mol Biol. 2020 Nov 6;43(4):e20200027. doi: 10.1590/1678-4685-GMB-2020-0027 (PMC7649911; doi:10.1590/1678-4685-GMB-2020-0027)
Supplement: Supplementary file 1 [file 1415-4757-GMB-43-4-e20200027-s1.pdf]

Genetics and Molecular Biology
https://doi.org/10.1590/1678-4685-GMB-2020-0027

Supplementary Material to "Molecular cytogenetic analyses reveal extensive chromosomal rearrangements and novel B chromosomes in *Moenkhausia* (Teleostei, Characidae)"

Table S1 – List of primers used in PCR amplifications.

Region		Primers	Sequence (5′ → 3′)	Reference
Histone	**H1**	H1F	*forward*: ATGGCAGAARYCGMCCAG	Hashimoto *et al*., (2011)
		H1R	*reverse*: TACTTCTTCTTGGGSGCTGC	
DNAr	**18S**	18SF	*forward*: GTAGTCATATGCTTGTCTC	White *et al*., (1990)
		18SR	*reverse*: TCCGCAGGTTCACCTACGGA	
	**5S**	5SF	*forward*: TCAACCAACCACAAAGACATTGGCAC	Pendas *et al*., (1994)
		5SR	*reverse*: TAGACTTCTGGGTGGCCAAAGGAATCA	
snDNA	**U2**	U2F	*forward*: ATCGCTTCTCGGCCTTATG	Bueno *et al*., (2013)
		U2R	*reverse*: TCCCGGCGGTACTGCAATA	
Telomeric	**Tel**	TelF	*forward*: (TTAGGG)n	Ijdo *et al*., (1991)
		TelR	*reverse*: (CCCTAA)n	
