# Supplementary material for: Molecular cytogenetic analyses reveal extensive chromosomal rearrangements and novel B chromosomes in Moenkhausia (Teleostei, Characidae)
Source: Genet Mol Biol. 2020 Nov 6;43(4):e20200027. doi: 10.1590/1678-4685-GMB-2020-0027 (PMC7649911; doi:10.1590/1678-4685-GMB-2020-0027)
Supplement: Supplementary file 2 [file 1415-4757-GMB-43-4-e20200027-s2.pdf]

Genetics and Molecular Biology
https://doi.org/10.1590/1678-4685-GMB-2020-0027

Supplementary Material to "Molecular cytogenetic analyses reveal extensive chromosomal rearrangements and novel B chromosomes in *Moenkhausia* (Teleostei, Characidae)"

Table S2 – Observed variation in the number of B chromosomes in *Moenkhausia*. F = female; M = male.

Species / Locality	Voucher	Sex	Cells with B chromosome					Total
			0B	1B	2B	3B	4B	
Moenkhausia forestii / **Ribeirão do Sapo**								
	564	F	21	–	–	–	–	21
	565	F	28	–	–	–	–	28
	577	F	12	–	–	–	–	12
	1357	M	13	5	4	–	–	22
	1358	M	10	–	9	8	–	27
	1359	F	5	5	–	–	–	10
	2103	M	13	1	–	–	–	14
	2109	F	9	1	–	–	–	10
	2110	F	16	4	1	1	–	22
	2111	F	12	1	–	–	–	13
	2166	F	5	21	–	–	–	26
	2168	M	9	–	–	–	–	9
M. oligolepis / **Corredeira Stream**								
	1361	M	47	3	2	–	–	52
	1362	M	7	7	2	–	1	17
	1363	F	5	–	–	–	–	5
	1364	F	49	–	–	–	–	49
	1365	F	12	–	–	–	–	12
	1366	M	15	7	5	2	7	36
	1367	M	14	–	–	–	–	14
	2149	F	13	–	–	–	–	13
M. oligolepis / **Xapuri River**								
	74836	M	21	10	–	–	–	31
	74839	F	9	3	2	–	–	14
	74840	F	16	–	–	–	–	16
	74863	F	31	1	–	–	–	32
	74864	F	21	–	–	–	–	21
	74865	M	12	2	1	–	–	15
	74866	M	5	6	–	–	–	11

Genetics and Molecular Biology
https://doi.org/10.1590/1678-4685-GMB-2020-0027

Species / Locality	Voucher	Sex	Cells with B chromosome					Total
			0B	1B	2B	3B	4B	
M. oligolepis / **Sangue River**								
	1438	F	8	–	–	–	–	8
	1439	F	10	–	–	–	–	10
	1440	M	4	–	–	–	–	4
	1441	F	2	–	–	–	–	2
	1442	M	30	3	7	5	–	45
	1443	M	19	3	–	–	–	22
	1444	M	14	–	–	–	–	14
